# More than One Way of Being a Moa: Differences in Leg Bone Robustness Map Divergent Evolutionary Trajectories in Dinornithidae and Emeidae (Dinornithiformes)

**DOI:** 10.1371/journal.pone.0082668

**Published:** 2013-12-18

**Authors:** Charlotte A. Brassey, Richard N. Holdaway, Abigail G. Packham, Jennifer Anné, Philip L. Manning, William I. Sellers

**Affiliations:** 1 Faculty of Life Sciences, University of Manchester, Manchester, United Kingdom; 2 School of Biological Sciences, University of Canterbury, Christchurch, New Zealand; 3 School of Earth, Atmospheric and Environmental Science, University of Manchester, Manchester, United Kingdom; Friedrich-Schiller-University Jena, Germany

## Abstract

The extinct moa of New Zealand included three families (Megalapterygidae; Dinornithidae; Emeidae) of flightless palaeognath bird, ranging in mass from <15 kg to >200 kg. They are perceived to have evolved extremely robust leg bones, yet current estimates of body mass have very wide confidence intervals. Without reliable estimators of mass, the extent to which dinornithid and emeid hindlimbs were more robust than modern species remains unclear. Using the convex hull volumetric-based method on CT-scanned skeletons, we estimate the mass of a female *Dinornis robustus* (Dinornithidae) at 196 kg (range 155–245 kg) and of a female *Pachyornis australis* (Emeidae) as 50 kg (range 33–68 kg). Finite element analysis of CT-scanned femora and tibiotarsi of two moa and six species of modern palaeognath showed that *P. australis* experienced the lowest values for stress under all loading conditions, confirming it to be highly robust. In contrast, stress values in the femur of *D. robustus* were similar to those of modern flightless birds, whereas the tibiotarsus experienced the highest level of stress of any palaeognath. We consider that these two families of Dinornithiformes diverged in their biomechanical responses to selection for robustness and mobility, and exaggerated hindlimb strength was not the only successful evolutionary pathway.

## Introduction

Before their rapid extinction coinciding with the arrival of Polynesian colonists [1], New Zealand's moa (Dinornithiformes) included some of the largest palaeognath birds, ranging in size from <15 kg to >200 kg. Recent genetic [2], radiocarbon [3], and stable isotope studies [4] have illuminated moa evolution, palaeogeography, and palaeoecology. Yet the most striking feature of dinornithiform biology, the immense range in body size and limb morphology between families (Megalapterygidae; Dinornithidae; Emeidae) and species and their resulting biomechanics, remain poorly understood. Stress levels within the extremely robust legs of the emeid *Pachyornis elephantopus* are predicted to have remained low during locomotion [5], with unusually high safety factors (the ratio of failure strength to the maximum stress it is likely to encounter) and poor running ability inferred in this species [6], [7]. Yet the more gracile giant moa (two species of *Dinornis*, which comprise the Dinornithidae) is reconstructed as being proficiently cursorial [8].

Estimation of safety factors and running speeds requires reliable values for body mass. Previous attempts at predicting moa body mass have favoured linear regression techniques [9], [10]. Yet the very nature of their unusually proportioned limbs makes mass estimation based on single linear dimensions problematic. This paper applies a volume-based mass estimation technique to two representative moa species, from the two families with most divergent morphologies, *Dinornis robustus*, the larger South Island dinornithid, and *Pachyornis australis*, the smaller of the two South Island emeids. *D. robustus* occupied the widest range of habitats of any moa, including lowland dry forests and shrublands, rainforests, subalpine shrublands and fellfields, whereas during the Holocene *P. australis* was confined to subalpine shrublands and fellfields where it was sympatric with *D. robustus* and *Megalapteryx didinus*.

To perform a comparative biomechanical analysis of skeletal elements, it is first necessary to derive a value for applied load for each model. Typical loads can be estimated as a multiple of the force acting on the skeleton due to gravity, and to calculate this we need to know the living body mass of the animal. As noted above, the extreme morphologies of moa long bones make body mass estimates for moa based on linear measurements unreliable. Here, we estimate moa body mass using a whole body volume technique. Subsequently we undertake a sensitivity analysis to quantify the effect of model reconstruction upon moa body mass estimates. We hypothesised that our volumetric technique would yield lower body mass estimates than those based on the diameter or circumference of the femur or tibiotarsus, given the unusual breadth of dinornithiform limb bones. This would therefore yield different estimates of the loads the bones had to carry, and the limitations on those loads.

We then compared the biomechanics of modern ratite and moa hind limbs bones using finite element analysis. Finite element analysis is a computerised technique in which a digital model is divided into a series of elements forming a continuous mesh. Material properties, boundary constraints and load conditions are applied to the model, and the resulting stresses and strains during loading are calculated. Previous biomechanical analyses of moa hind limbs have relied upon simplified beam theory models [5], [11], in which complex organic structures are simplified into slender beams. However, in a broad sample of morphologically diverse mammal and bird long bones, the errors introduced into stress calculations resulting from this simplification have been shown to be neither consistent in magnitude nor direction [12]. Factors such as shaft curvature, low values of aspect ratio (length/diameter) and variations in cortical wall thickness are characteristic of organic structures such as long bones, yet these are typically unaccounted for in simple beam equations [12]. However, finite element analysis allows the complex 3D geometry of bones to be incorporated into stress equations, and with access to computed tomography (CT) facilities becoming cheaper and easier, it is now feasible to generate a larger comparative dataset of 3D models on which to perform biomechanical analyses.

Here we use our new body mass estimates and finite element models for moa to compare limb bone robustness of these Dinornithiformes to those of modern palaeognaths and discuss the results in the context of habitat preferences and locomotor modes. Given the reputation of moa as being ‘robust’ (*Dinornis robustus*, the etymon of robust terrible bird; and *Pachyornis australis*, the southern thick/stout bird), we might hypothesise that their limb bones ought to experience lower levels of stress than modern palaeognaths when loaded under equivalent conditions. The present study is the first attempt to quantify such biomechanical variation in the different lineages of this order of large birds.

## Materials and Methods

### Convex hull calibration on modern ratites

**Figure 1:**
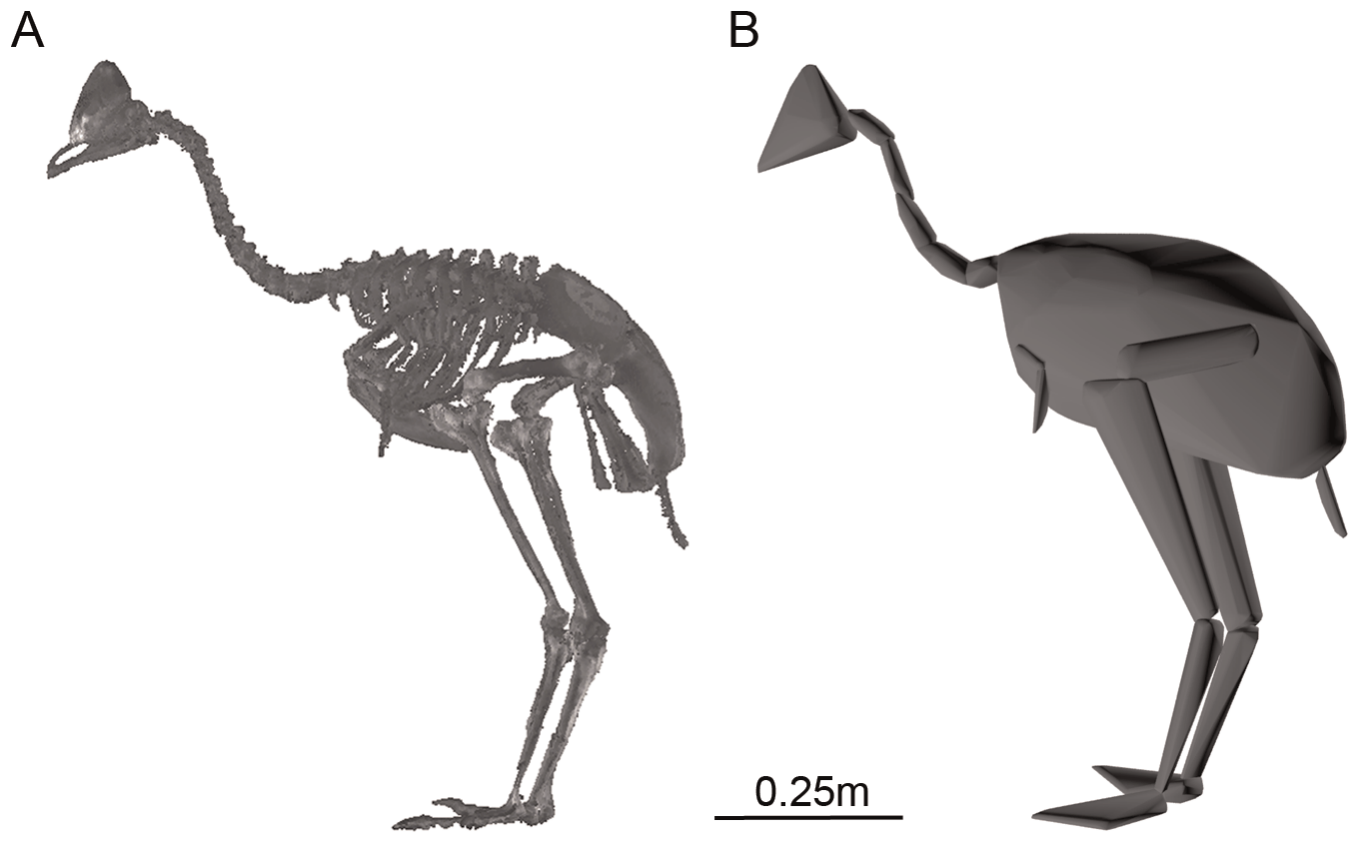
The convex hulling process (*a*) Point cloud data for *C. casuarius* derived from LiDAR (light radar) scanning; (*b*) convex hulls of each body segment.

All skeletal material included in this study was accessed with the permission of the relevant museum (University Museum of Zoology, Cambridge; National Museums Scotland, Edinburgh; Museum of New Zealand, Te Papa Tongarewa) and reside within their permanent collections. The mounted skeletons of modern species of ratites were scanned using a Z+F Imager 5010 LiDAR (light radar) scanner at the University Museum of Zoology, Cambridge (UMZC) (see Table 1). Reconstructions were carried out in Z+F LaserControl and Geomagic Studio v.12 (Geomagic, USA), and point clouds representing individual skeletons were isolated (see Figure 1a). Each individual was then subdivided into functional units: feet (phalanges), hand (metacarpals and phalanges), metatarsus, shank (tibiotarsus), thigh (femur), distal wing (radius and ulna), proximal wing (humerus), trunk (pelvis, ribs, sternum, sternal ribs), neck and skull. The neck was subdivided into 5 parts to ensure a tight-fitting convex hull around its curvature. Each functional unit was saved as a point cloud, and the minimum convex hull calculated using the qhull command of MATLAB (MathWorks, USA) (see Figure 1b) from which enclosed volumes were calculated. A convex hull is defined as the smallest convex object that can be fitted around selection of points, and in practical terms can be visualised as stretching a rubber sheet around the given set of points.

**Table 1:** Convex hull specimen list and sources of body mass.

species	accession no.	volume (m^3^)	*M*_b_ (kg)	*M*_b_ source	Scaling equation	*x*	*n*
*Struthio camelus*	UMZC374	0.0717	60.7	[48]	*y* = 0.374log*x*-log1.259	femur length	15
*Casuarius casuarius*	UMZC371.D	0.0172	27.0	[49], [50]	*y* = 4.69*x*+189.6	tibiotarsal length	3
*Dromaius novaehollandiae*	UMZC363	0.0214	20.06	*	*y* = 6.35*x*+92.6	femur length	3
*Rhea americana*	UMZC378.99	0.0177	16.3	[51], [52] **	*y* = 10.21*x*+140.2	tibiotarsal length	3
*Rhea pennata*	UMZC378ki	0.0159	14.9	[51], [52] **	*y* = 10.21*x*+140.2	tibiotarsal length	3
*Apteryx australis*	UMZC378.A	0.00106	2.96	[53]	*y* = 3.6*x*+20.33	femur circumference	30
*Apteryx australis lawryi*	UMZC378.55	0.00137	2.41	[53]	*y* = 3.6*x*+20.33	femur circumference	30

*M*_b_) was estimated for the convex hull individuals by first generating species-specific least squares regressions of known body mass against a linear metric from the hind limb as reported in the literature. Body mass (

*Dromaius novaehollandiae* femoral length against body mass derived from carcasses of known body mass from the University of Manchester. Regression equation of

*Rhea spp.* tibiotarsal length against body mass generated from previously published raw data and one carcass from the University of Manchester. Regression equation of

Unfortunately, associated body masses were not available for the mounted museum skeletons. We measured linear dimensions (femur and tibiotarsal length, and midshaft circumference) directly from the skeletons. Body masses were then estimated using species-specific regression equations, derived either from the literature or generated by the authors based on published raw values (see Table 1). Literature-derived values for body mass were then regressed against convex hull volume in R [13]. Unlike previous studies [14], convex hull volume was not converted to a minimum mass by multiplying by density. Values for avian body density are sparse in the literature (see later Discussion), and frequently refer to plucked carcasses. Furthermore, post-mortem collapse and infilling of air sacs with fluid most likely causes a significant increase in body density relative to live birds. However it is likely that the body density of ratites does not vary much between species. Convex hull volume (*cvol*) was therefore immediately regressed against literature mass to avoid introducing further uncertainty into the analysis. A summary of the existing empirical data for avian body density is included later in the discussion.

Regression analyses were carried out in the R package ‘smatr’ [15] using both Type-I (least squares linear regression, LR; linear regression through the origin, LRO) and Type-II (major axis regression, MA; standard major axis regression, SMA) line-fitting techniques on untransformed data which met the requirements of normality and homoscedasticity. Linear regression, MA and SMA are all least-squares line-fitting methods, but differ in the direction in which distances between the line and data points are measured. For more details regarding their application, see Warton et. al. [16].

### Reconstruction of moa skeletons and mass estimation

The two moa individuals were selected from the collection of the Museum of New Zealand Te Papa Tongarewa on the basis of possessing pelves and complete hindlimb skeletons. The specimen of *P. australis* (S.27896) lacked several ribs. The South Island giant moa (*Dinornis robustus*) specimen (S.34088) lacked several vertebrae and the skull; the skull of a second large *D. robustus* individual (S.34089) was therefore included. Skeletal elements were digitally remounted in accordance with recent reconstructions, in which the vertebral column is bent forward and downward into a loop and the head is held only slightly higher than the top of the pelvis [9]. As the *D. robustus* specimen lacked many vertebrae, two additional vertebrae were added to the reconstructed vertebral column of *P. australis* (due to differences in vertebral formulae between Emeidae and Dinornithidae [9]) which was subsequently scaled up geometrically to fit the larger *D. robustus*.

The process of digitally remounting skeletons from disarticulated elements introduces a degree of uncertainty into our mass predictions. In particular, the positioning of the sternum and ribs defined the volume of the convex hulled trunk, which itself contributed most to the total volume of the bird. In both moa specimens, several thoracic and sternal ribs lacked their ventral extremities or were absent. The convex hulling process was therefore repeated with the sternum in higher (*cvol*_min_) or lower (*cvol*_max_) positions dorsoventrally, to allow for uncertainty in the positioning of the sternum in the living bird. The final confidence intervals for our moa mass estimates were therefore calculated by inserting the values for *cvol*_max_ and cvol_min_ into the convex hull equation, using the upper and lower values of the prediction interval respectively.

### Computed tomography (CT)

The 3D models forming the basis of our finite element analysis were derived from CT scans of femora and tibiotarsi. In most instances, femora and tibiotarsi were acquired from the bird collection of the National Museum of Scotland, Edinburgh (Table 2). All museum-sourced specimens were deemed skeletally mature (on the basis of plumage records and surface rugosity of the femoral and tibiotarsal shaft [17]), and were free of pathologies. However, for the emu (*Dromaius novaehollandiae*) and rhea (*Rhea americana*) hindlimb “bones” were extracted from whole carcass CT scans of the individuals. The emu was euthanised at an age of 10 weeks, and should therefore be considered to be subadult ([18] and see later Discussion). In each specimen, the stylopodium and zeugopodium were sourced from the same individual, and whenever possible, from the same limb. For the emu and rhea, body mass (*M*_b_, kg) was recorded post-mortem. For museum specimens, associated body masses were not available and values were therefore assigned using literature species-specific scaling equations (see Table 2).

**Table 2:** Finite element analysis specimen list and sources of body mass.

species	accession no.	*M*_b_ (kg)	*M*_b_ source	Scaling equation	*x*	*n*	*F* (N)
*Struthio camelus*	NMS 1930.15.1	100	[48]	*y* = 0.374log*x*-log1.259	femur length	15	980.6
*Casuarius unappendiculatus*	NMS 1995.119.1	49.8	[49], [50]	*y* = 4.69*x*+189.6	tibiotarsal length	3	488.1
*Dromaius novaehollandiae*	-	16.05	-	carcass weight	-	-	157.4
*Rhea americana*	-	7.85	-	carcass weight	-	-	77.01
*Apteryx haasti*	NMS 1913.48	2.80	[53]	*y* = 3.6*x*+20.33	femur circumference	30	27.47
*Tinamus solitarius*	NMS PS276/04	1.46	[53]	*y* = 8.17*x*+9.673	femur circumference	28	14.32

Table 1. For *Dromaius novaehollandiae* and *Rhea americana*, body mass was recorded directly from the carcass. *F*, total force applied to the finite element model in Newtons. Body mass estimated for the finite element analysis specimens using the same species-specific regressions of known body mass against a linear metric from the hind limb, as in

Small modern palaeognaths (*Tinamus solitarius, Apteryx haasti*) were scanned at the Henry Moseley X-ray Imaging Facility, University of Manchester (X-Tek HMX 225 Custom Bay, Nikon Metrology Ltd, UK) at a voxel spacing of 85–119 µm. *Rhea americana, Dromaius novaehollandiae, Casuarius unappendiculatus*, and *Struthio cameleus* were scanned in a helical CT scanner at the University of Liverpool Small Animal Teaching Hospital (Siemens SOMATOM Volume, Germany) at pixel spacings of 270–867 µm and slice thicknesses between 1–1.5 mm. The two dinornithiform skeletons were scanned by Pacific Radiology (Southern Cross Hospital, Wellington, New Zealand) in a helical CT scanner (BrightSpeed, GE Healthcare, USA) at a pixel spacing of 320–977 µm and a slice thickness of 0.625 mm.

### Estimating hind limb robustness using finite element analysis

Hindlimb bone scans were segmented in Avizo v.7.1 (VSG Inc., USA), and periosteal and endosteal surfaces were isolated and repaired in Geomagic v.12 (Geomagic, USA). OBJ files were converted into SAT file format using Form•Z (AutoDesSys®) and imported into Abaqus (Simula®, USA) in which finite element analysis was undertaken. The finite element analysis carried out in this study follows the methodology of Brassey et al [12]. An instance was created in Abaqus containing both parts, and a Boolean operation used to subtract the endosteal part from the periosteal part to create a hollow bone model. A homologous value for Young's modulus of 19 GPa and Poisson's ratio of 0.3 were assigned to all models [19]. Hollow bone parts were meshed using a built-in Delaunay meshing algorithm within Abaqus.

The total number of elements in each model was set at c. 1 million (range, 960,059–1,030,551). A previous sensitivity analysis found stress values predicted by finite element analysis converged above 800,000 elements in a broad sample of vertebrate long bones [12], and a value of 1 million was chosen to ensure convergence. The same study compared stress values between 4-node linear tetrahedral meshes and 10-node quadratic tetrahedral meshes, and found stress values to converge in models exceeding 200,000 elements. C3D10 tetrahedra are computationally more expensive [20], and C3D4 tetrahedral meshes were therefore used throughout this study.

**Figure 2:**
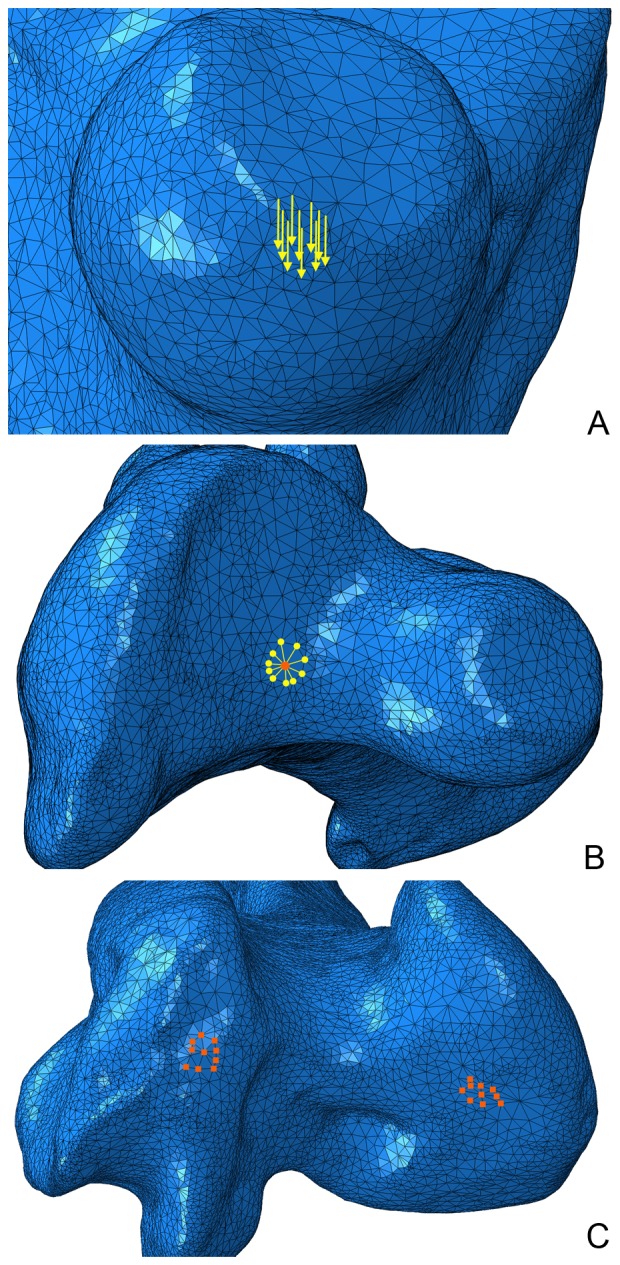
Loading regimes for finite element analysis of *Dinornis* femur (*a*) Medial view of femoral head, yellow arrows originate from the nodes to which force is applied. The direction of force is aligned parallel to the long axis of the bone, i.e. loading in compression. (*b*) Dorsal view of the proximal femoral epiphysis. Orange dot represents constrained control point, and is surrounded by 10 yellow dots representing the nodes to which torsion is applied via the kinematic coupling. (*c*) Ventral view of the distal femoral condyles. Orange squares represent nodes subject to encastre boundary conditions.

Models were loaded under combined compression and bending (0–90° of vector orientation in the parasagittal plane) and torsion. Total load applied was equivalent to 10% of body mass. A small multiple of body mass was chosen to ensure that total strain values were small, and deformation remained within the linear elastic region (as in [12], [21]). For femora, the applied force was spread across 10 adjacent nodes on the medial surface of the femoral head (Figure 2a). For tibiotarsi the load was applied on 10 nodes across the intercondylar eminence. To simulate combined compressive-bending loading, force was initially applied parallel to the principal axis of the bone, and then the load vector incrementally modified from 10–90° from the principal axis.

All models were also loaded under axial torsion. The condyles of the distal epiphyses were constrained in all three directions, and a constraint control point (CP) created on the proximal epiphyses. For femoral torsion, the moment was not applied on the femoral head: rather, the CP was located on the proximal surface between the head and the major trochanter, corresponding to the location at which the bone's longest principal axis emerged at the surface (Figure 2b) [12]. This orientation ensured that torsion was about the long axis of the femur. The CP was constrained in three directions, and a kinematic coupling created between 10 nodes surrounding the CP, and the CP itself (Figure 2c). A torsional moment about the bone's principal axis was applied at the CP (proportional to 10% of body mass), and transmitted via kinematic coupling to the load surface. For all loading regimes, 20 nodes on the surface of the distal epiphyses were constrained using the ‘encastre’ boundary condition (Figure 2c).

A linear elastic analysis was carried out on all models, and equations solved using Gaussian elimination. Zones of stress concentration are likely to occur at fixed boundaries as a result of reaction forces at constrained nodes. Stress values were recorded therefore from the midshaft of the bone models, a considerable distance from the fixed boundary nodes. For all loading regimes, the greatest value of Von Mises stress located on the periosteal surface at midshaft (σ_vm_) was extracted. The effect of sternal position on stress estimates in the dinornithiform individuals was investigated by substituting minimum and maximum values for moa body mass in the analysis. Point cloud and CT data are available from animalsimulation.org.

## Results

### Moa Body Mass Estimates

**Figure 3:**
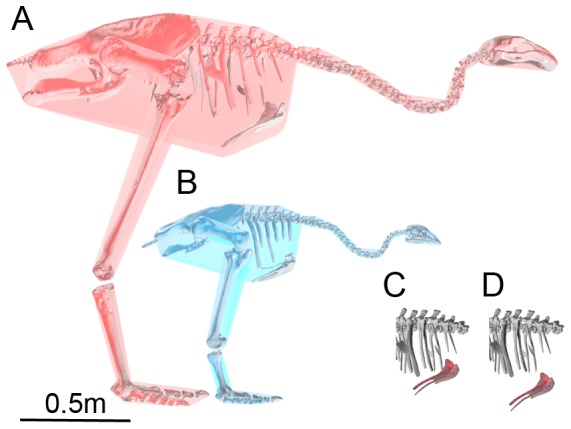
Moa convex hulls (*a*) *Dinornis robustus* (S.34088/89) reconstruction of convex hulls; (*b*) *Pachyornis australis* (S.27896) (*a* and *b* are to the same scale); (*c*) and (*d*) show different positions of the sternum in *D. robustus*.

**Figure 4:**
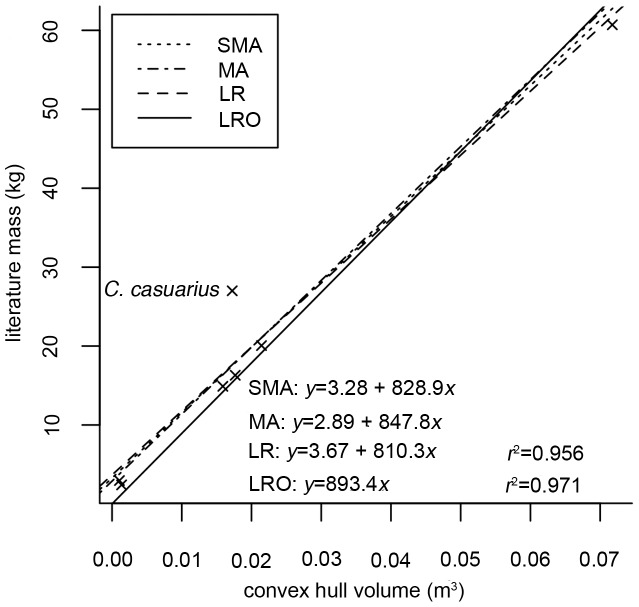
The relationship between convex hull volume and literature values for mass in extant ratites. LR, linear regression; SMA, standardized major axis regression; MA, major axis regression; LRO, linear regression forced through the origin.

Individual body segment volumes and total convex hull volumes are given in Table 3. Figure 3 shows the convex hull reconstructions calculated for the moa specimens. The relationship between convex hull volume and body mass in extant ratites is given in Figure 4. All regression techniques produce very similar answers, were all highly statistically significant (*p*<0.005) and had high correlation coefficients (*r* ^2^>0.95). Following the logic of Sellers et al. [14], we also applied the LRO (linear regression through the origin) equation (*y* = 893.4*x*, 95% CI = 740–1048, *p* = 0.003, *r* ^2^ = 0.97) to estimate the live mass of our dinornithiform individuals. LRO arguably makes better biological sense as an individual with zero volume must have zero mass, and Type-I regressions are recommended where the regression model will be used in a predictive capacity [22]. The data point for *C. casuarius* appeared to be an outlier (Figure 4). This probably resulted from the uncertainty in the body mass estimate for *C. casuarius*, as there are few published accounts of individual cassowary limb proportions and their corresponding body mass. However, removing the data point had no significant effect on the value of the slope (with *C. casuarius b* = 893.4, without *C. casuarius b* = 861.4, *p* = 0.52). Predicted masses, including the results of the sensitivity analyses, are shown in Table 4: the average mass for *D. robustus* was 196 kg (95% confidence interval 155–245 kg), and that for *P. australis* 50 kg (95% confidence interval 33–68 kg).

**Table 3:** Moa convex hull volumes and body segment volumes.

	*cvol* (m^3^)	
	*D. robustus*	*P. australis*
Trunk	0.1595 ^(0.152–0.172)^	0.0360 ^(0.033–0.039)^
Femora	0.0111	0.0040
Tibiotarsi	0.0212	0.0084
Tarsometatarsii	0.0118	0.0045
Toes	0.0066	0.0020
Neck	0.0030	0.0006
Skull	0.0055	0.0007
Total	0.2187	0.0562

Trunk values include minimum and maximum volumes defined by shifting the sternum dorsoventrally. Segment values consist of the sum total of left and right elements.

**Table 4:** Body mass estimates of moa individuals.

	mass (kg)	95% prediction interval (kg)
*D. robustus*		
*cvol*	195.7	159.8–231.5
*cvol*_min_	189.4	**154.5**–224.3
*cvol*_max_	207.3	169.5–**245.0**
*P. australis*		
*cvol*	50.3	35.2–65.4
*cvol*_min_	47.9	**32.8**–62.5
*cvol*_max_	52.9	37.5–**68.2**

*cvol*, mean convex hull; *cvol*_max_, maximum convex hull volume with sternum positioned ventrally; *cvol*_min_, minimum convex hull volume with sternum positioned dorsally. Bold values indicate minimum and maximum body mass values inserted into FE sensitivity analysis.

### Finite Element Analysis

**Figure 5:**
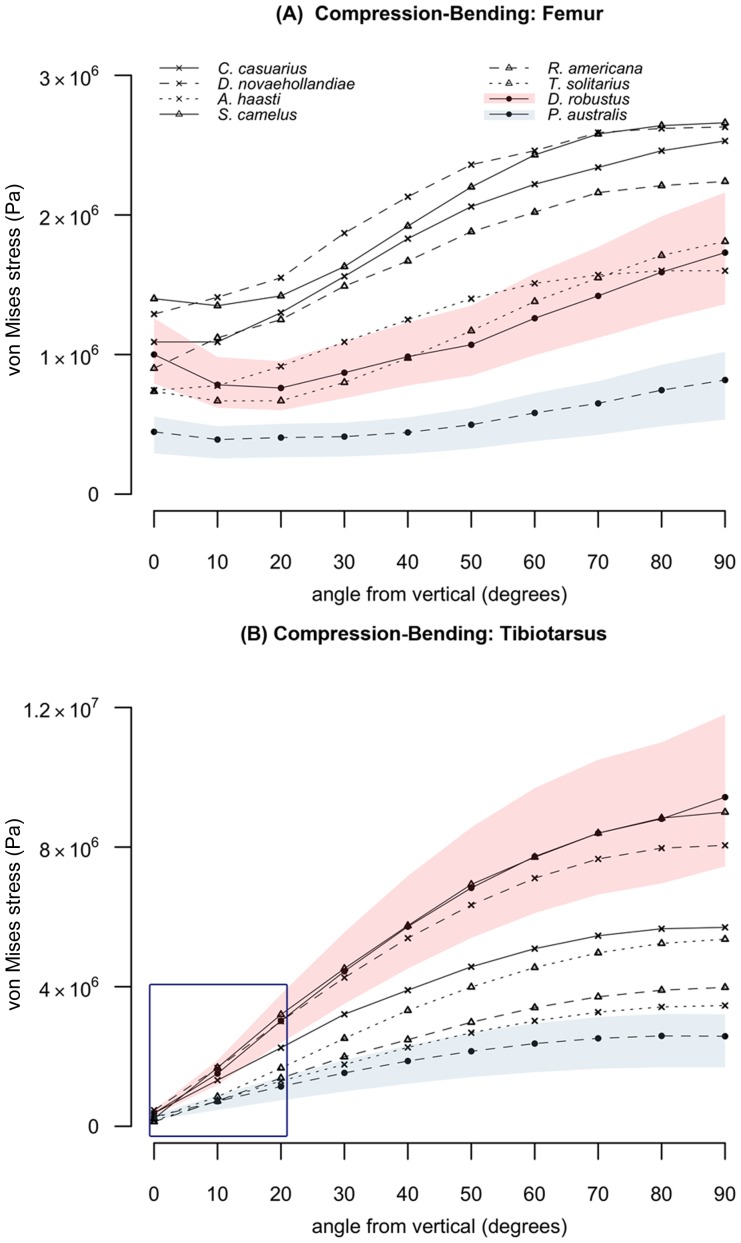
Finite element analysis results. Combined compression-bending results for the femur (a) and tibiotarsus (b). Values represent maximum von Mises stress (Pa) recorded at the midshaft of the bone. Pink and blue shaded areas represent the range of stress values estimated by finite element analysis when incorporating maximum and minimum values for body mass in *D. robustus* and *P. australis* respectively. Area enclosed by dark blue box is expanded in Figure 6.

**Figure 6:**
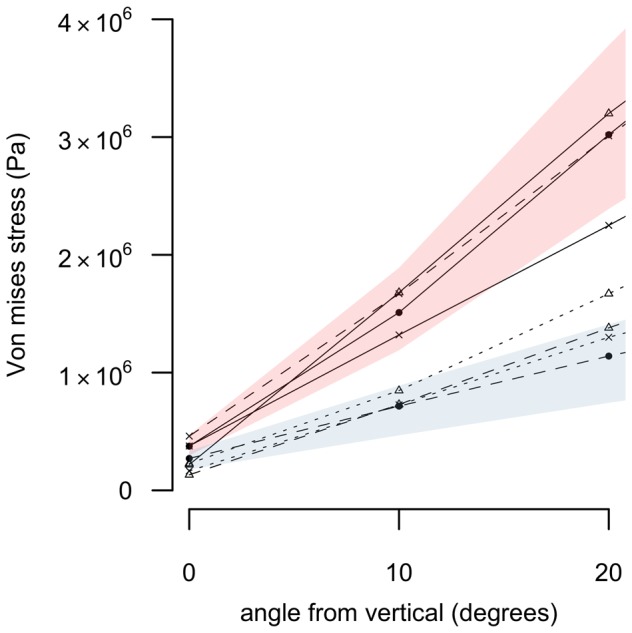
(inset of Figure 5) Combined compression-bending of the tibiotarsus between 0–20° from vertical. Values represent maximum von Mises stress (Pa) recorded at the midshaft of the bone. Legend as in figure 5.

**Figure 7:**
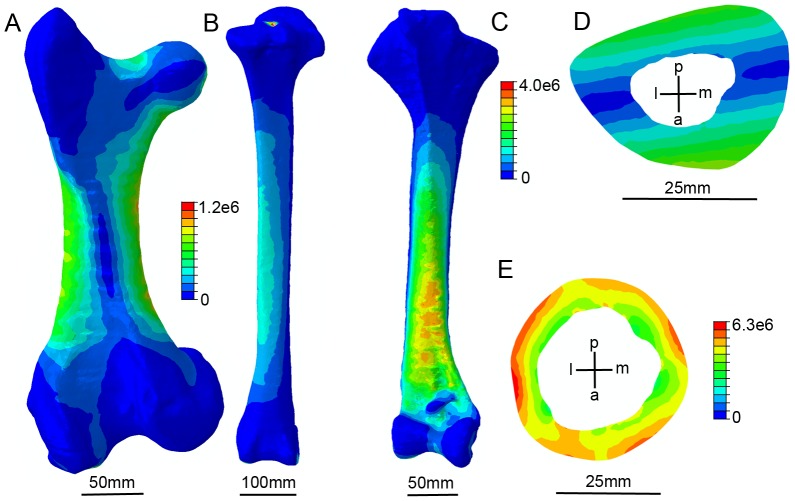
The distribution of Von Mises stress within moa finite element models. (*a*) *Dinornis* femur loaded in compression (0° from the longest principal axis) experienced a significant degree of bending due to off-axis application of force on the femoral head. (*b*) *Dinornis* tibiotarsus experienced lower values of σ_vm_ under compression, and underwent less bending due to application of forces on the intercondylar eminence. (*c*) *Pachyornis* tibiotarsus loaded in bending (90° from the longest principal axis). σ_vm_ increases towards the fixed end of the beam, with localised areas of stress related to variations in cortical wall thickness. (*d*) Slice through midshaft of *c.* Values of σ_vm_ are highest at the extreme compressional and tensional cortices with a neutral axis of lowest stress values running between. (*e*) Slice through midshaft of *Pachyornis* femur loaded in torsion. Stress values increase radially from the endosteal to periosteal surface, with the highest stresses located in regions where cortical wall thickness is at a minimum. For (*d*) and (*e*), bone orientation is indicated by coordinate system (a–p, anteroposterior; m–l, mediolateral).

Maximum Von Mises stresses (σ_vm_) when femora and tibiotarsi were loaded from compression (0°) to cantilever bending (90°) and torsion are shown in Figures 5 and 6. The location of peak stresses within finite element models typically correspond to those predicted by simple beam models. However both femora experienced induced bending when loaded in compression (Figure 7a). This can partially be explained by curvature-induced bending [12], but for femora it is particularly so because of the off-axis application of force on the femoral head. The avian tibiotarsus is typically less curved than the femur [23], and the load was applied across the intercondylar eminence. For these reasons, the dinornithiform tibiotarsi experienced lower bending stresses when loaded parallel to their long axes (Figure 7b).

Under bending, the distribution of stresses in finite element models closely matched the predictions of a fixed cantilever beam model. Von Mises stress increased incrementally towards the fixed end (Figure 7c), with a band of low stress values (neutral plane) located between the compressional and tensional cortices (Figure 7d). When loaded in torsion, Von Mises stress increased radially from the endosteal to periosteal surface, with the highest values of σ_vm_ located in areas of minimum cortical wall thickness (Figure 7e).

The lowest values of σ_vm_ were found in the femur and tibiotarsus of *P. australis* (Figure 5*a,b*), with confidence intervals not overlapping those of any other palaeognath under high levels of bending. The stress values measured in *D. robustus* femur were intermediate, overlapping those of *A. haastii* and *T. solitarius*. The *D. robustus* tibiotarsus exhibited the highest values for σ_vm_ under bending, but with values overlapping those of *S. camelus* and *Dr. novaehollandiae*. When the tibiotarsus of *D. robustus* was loaded predominantly in compression, however, σ_vm_ values were lower than those for *S. camelus* and *Dr. novaehollandiae* (Figure 6). Under torsion (Table 5), both dinornithiforms exhibited low values of σ_vm_, with their confidence intervals failing to overlap those of modern species. The *P. australis* tibiotarsus was significantly less stressed than that of *D. robustus* under torsion.

**Table 5:** Finite element analysis results for torsional loading.

	von Mises stress (Pa)	
	femur	tibiotarsus
*C. casuarius*	2.21×10^7^	3.07×10^7^
*D. novaehollandiae*	3.33×10^7^	4.17×10^7^
*A. haasti*	3.06×10^7^	4.67×10^7^
*S. camelus*	1.96×10^7^	2.98×10^7^
*R. americana*	2.92×10^7^	3.20×10^7^
*T. solitarius*	5.51×10^7^	1.14×10^8^
*D. robustus*	9.45×10^6^	2.07×10^7^
(mass-dependent range)	7.46×10^6^–1.18×10^7^	1.63×10^7^–2.59×10^7^
*P. australis*	6.30×10^6^	1.09×10^7^
(mass-dependent range)	4.12×10^6^–7.84×10^6^	7.14×10^6^–1.36×10^7^

Values represent maximum von Mises stress (Pa) recorded at the midshaft of the bone. For the two moa species, the range of von Mises stresses based on minimum and maximum body mass estimates (Table 4) is also presented.

## Discussion

### Body mass estimates

Our estimate of 195 kg for the body mass of *D. robustus* was just over 80% of the estimate of 238 kg [10] based on the averaged femoral circumference of seven *D. robustus* individuals calculated from a ratite-specific regression. However, our maximum range calculated through sensitivity analyses (155–245 kg) was considerably narrower than confidence intervals calculated from the linear regression (164–346 kg). Applying palaeognath-specific scaling equations of femoral and tibiotarsal length and diameter against body mass [24], mass estimates for this specimen of *D. robustus* range between 226–517 kg depending upon the metric used (Table 6). Our volume-based mass predictions are therefore lower than those produced by linear regression techniques.

**Table 6:** Moa body mass estimates (kg) and 95%CI based derived from palaeognath-specific regressions of femoral and tibiotarsal metrics published by Cubo and Casinos [24].

	*Dinornis robustus* (kg)	*Pachyornis australis* (kg)
Femur length	488 (357–709)	144 (115–187)
Femur diameter (AP)	237 (200–287)	115 (100–133)
Femur diameter (ML)	289 (231–384)	111 (95–137)
Tibiotarsus length	517 (382–738)	115 (74–107)
Tibiotarsus diameter (AP)	226 (178–296)	94 (79–114)
Tibiotarsus diameter (ML)	311 (254–406)	124 (107–152)

Our estimate of 50 kg (range 33–68 kg) for the Pleistocene-aged *P. australis* is also lower than the species mean of 116 kg (95% CI 86–158 kg) predicted on the basis of ratite femoral circumference of all Pleistocene-aged individuals [10]. Yet, our estimate falls within the range (44–90 kg) of values for *P. australis* calculated from femoral length for birds of that period [9]. Applying the ratite scaling equations derived by Cubo and Casinos [24] mass estimates range between 94–144 kg, again being considerably higher than our volume-based mass prediction (Table 6).

A major advantage of volume-based reconstructions is the inclusion of information from the whole skeleton [14]. When dealing with skeletal extremes, such as the hyper-robust femora of *Pachyornis*, mass predictions based on a single linear dimension can result in significant under- or over-estimations. Furthermore, when a range of scaling equations are derived from single linear dimensions, it leads to uncertainty in which dimension is most appropriate to use as a mass predictor. As can be seen in Table 6, applying a mass prediction equation based on femoral length results in significantly higher estimates than those based on femoral diameter. In particular, the choice of ecologically or locomotorily specialized limbs is problematic when applying mass prediction equations to single elements. In contrast, volumetric approaches incorporate the maximum amount of information from a skeleton in one measure, avoid the single bone problem [14] when animals have unusual sized limbs and require no a priori assumption of which skeletal element ought to be used in the predictive equation.

Because the convex hull volume is the minimum possible volume, by taking the mean predicted mass of the moa models and their convex volumes, we estimated a maximum possible body density of 895 kg/m^3^ for the individuals. This compares to values ranging from 730 kg/m^3^ for a sample of flying birds [25], 894–968 kg/m^3^ for junglefowl and broiler chickens [26], 888 kg/m^3^ for an ostrich [27], 900 kg/m^3^ for a duck [28] and 937 kg/m^3^ for a goose [29]. These literature values were estimated using a variety of methodologies, and no single study has adequately dealt with the question of avian body density. Furthermore, the present analysis does not account for the presence or absence of gizzard stones in extant or extinct specimens. The total mass of gizzard stones may reach 1 kg in modern ostrich [30], whilst 5 kg of gastroliths have been found in association with a *Dinornis robustus* [31]. However given the mass estimates presented here, dinornithiform gastroliths likely contribute only 2–3% of total body mass.

### Finite element analysis results

Having generated predictions for the body mass of *D. robustus* and *P. australis* that were lower than published values, we incorporated these new estimates for *M*_b_ into the finite element analysis of the hind limb bones as a value for applied force. For every loading condition considered, values of σ_vm_ extracted from the finite element analysis were lowest in the leg bones of *P. australis* (Figure 5*a,b*), and this species is confirmed as having been extremely robust. Hyper-robustness of limbs could conceivably be an adaptation towards unpredictable loading conditions. Indeed, the ‘rough and tumble’ lifestyle of many birds has been put forward as an explanation as to why the hollow long bones of birds do not confirm to mechanical predictions for minimal mass [32]. *P. australis'* habitat range during the Holocene was restricted to subalpine regions of the northwest South Island, and robust limbs would have proved advantageous in upland environments with uneven terrain.

This does not explain the hyper-robustness of *P. australis* limbs however. Warm Holocene-like climatic conditions have been exceptional during the past 1 million years, with glacial conditions being the climatic norm [33]. As a species, *P. australis* occupied different altitude ranges as climate changed during glacials, interglacials and transitions, and spent most of its evolutionary history in lowland low-relief environments. Limb robustness in *P. australis* is therefore unlikely to be a specific adaptation to upland environments. Indeed, the larger sister-species *P. elephantopus* occupied lowland regions throughout the Quaternary despite appearing to possess even more robust limbs.

In contrast to *P. australis*, values of σ_vm_ in the legs of *D. robustus* were comparable to, or exceeded those of modern ratites under compression and bending (Figure 5*a,b*). Despite deriving a lower estimate of body mass, *D. robustus* is therefore reconstructed as a gracile ratite. *D. robustus* remains have been identified from a range of habitats spanning lowland forest, shrubland and subalpine locations, where it co-existed with *P. australis*. Alongside *M. didinus*, their bones are common in the same subalpine caves in northwest Nelson where Holocene *P. australis* remains are found, yet neither taxon exhibited the same degree of robustness seen in *P. australis*. Hindlimb robustness does not therefore appear to be correlated with habitat preference in diornithiforms, with the hyper-robust *P. australis* and relatively gracile *D. robustus* living sympatrically throughout the Holocene. Despite this spatiotemporal overlap, our sample of dinornithiforms exhibits greater variance in tibiotarsal robustness than that of modern ratite species spanning several continents and diverse habitats. An alternative hypothesis is therefore required to explain the disparity in moa hindlimb biomechanics.

The robustness of *P. australis'* hindlimbs may be associated with the evolution of different leg bone length proportions that characterise emeids compared to other moa and large palaeognaths. A distinguishing synapomorphy of the Emeidae is the relatively short tarsometatarsus, and the associated mediolateral expansion of this element and the distal tibiotarsus. Reducing the length of the ‘effective hindlimb’ (tibiotarsus plus tarsometatarsus) and increasing mediolateral width would result in increased resistance to lateral loading whilst limiting maximum stride length. The suite of modifications that resulted in the distinctive tarsometatarsal of emeids implies a divergence in locomotor capabilities or other habitual behaviours between *P. australis* and *D. robustus* whilst occupying the same habitat. To test the hypothesis that *P. australis* and *D. robustus* occupied distinct ecological niches whilst occupying the same habitat, future biomechanical analyses of Dinornithiformes would benefit from incorporating additional data regarding gastrolith, coprolite and bone stable isotopic composition as indicators of diet preference and territory range [34].

The distinction between *P. australis* and *D. robustus* is less pronounced during compressive-bending loading of the femur compared to the tibiotarsus. Under torsional loading of the femur, the stress values estimated from the sensitivity analysis of the moa individuals overlap considerably (Table 5). The avian femur is constrained to a subhorizontal posture at low to moderate speeds [35], and locomotor/behavioural specialisations within moa are played out via modifications to the tibiotarsus and tarsometatarsus. In a broad sample of modern birds, species with the highest predicted tibiotarsal safety factors under static bending included aerial hunters, hindlimb-propelled divers, and waders [23] rather than ground-dwelling galliformes and ratites. High safety factors in the tibiotarsus of modern birds do not reflect cursoriality, but are instead correlated with habitual behaviours such as prey capture or a preference for compliant substrates (both of which imply load unpredictability).

The emu individual included within our finite element analysis dataset was subadult at the time of euthanasia. As such, the stress values estimated using finite element analysis might not reflect those of a skeletally mature individual. The femur and tibiotarsus of the subadult emu experienced some of the highest values of σ_vm_ for modern ratites under combined compression-bending (Figure 5). A kinematic study of emu locomotion found significant ontogenetic increases in principal strain in the hind limb, despite negative allometric scaling of shaft curvature and constant relative limb loading throughout growth [36]. Higher values of σ_vm_ than those found in our emu individual might therefore be expected in fully adult individuals.

A homogeneous value for Young's modulus was applied to all ratite finite element models. The intra-element variation of material properties in vertebrate long bones have been discussed extensively elsewhere [37], and reported values for Young's modulus in avian bone vary significantly between species and between limb bones [38]. Furthermore, both the moa and kiwi have been found to possess bone histology atypical of most ornithurines, consisting of annual growth rings in their limb bones [39], [40]. By assigning a single value for Young's modulus across species, potential material effects that may contribute to total stiffness of the ratite hind limb are ignored. Furthermore we include a subadult emu in our sample, despite evidence to suggest ontogenetic variation in material properties across vertebrates [41]. In addition, the safety factor at which a limb bone operates is both a function of the experienced strain and the yield strain of the material. Here we assume that yield strain does not change and we directly compare stress values derived from our finite element models between species. Yet a weak, but highly significant, negative correlation does exist between yield strain and Young's modulus [42]. However the variation in Young's modulus and yield strain between bird species, skeletal elements and age groups has yet to be adequately described using a consistent material testing technique. As such, attempting to incorporate species-specific values into a comparative finite element analysis would currently act to increase uncertainty in estimated stress values and resulting safety factors. Therefore, the analysis presented here deals with the geometric differences between moa skeletons only, and the variability in elastic bone material properties and their subsequent effect on finite element analysis results will require further work (but see [21]).

Moa exhibited considerable divergence in their hindlimb morphology, and consequently biomechanical functionality, between families. Moa possessed a variety of adaptations to flightlessness, but only one of the three lineages – Emeidae – evolved more robust limb bones. Here we include only one representative from each of the Dinornithidae and Emeidae, and in effect carry out a two-species comparison. We therefore cannot conclude that the differences in limb robustness between moa families solely reflect alternative locomotor capabilities, but may also be associated with divergent life history strategies, physiologies, or separate evolutionary histories. In island giant species, an overreliance upon selection-based explanations (assuming biomechanics to be critical in all species) should be avoided. In a two-species comparative study, some degree of genetic differentiation is to be expected as a result of the speciation process and subsequent genetic drift alone, and therefore a more appropriate null hypothesis might have been that our two species ought to have been different as a result of their separate evolutionary histories, rather than no difference existing [43]. The New Zealand avifaunal fossil record is one of the best of the world for the Holocene and late Pleistocene [9], and the few moa fossils found to date earlier than the Pleistocene [44], [45] support the contention based on extensive genetic evidence, that the dinornithids and emeids split between 4–6 million years ago [46]. The two families therefore spent a considerable amount of time on separate evolutionary trajectories. However, in the absence of a detailed pre-Pleistocene fossil record, the pattern of morphological change within each genetic lineage throughout the Cenozoic remains unknown.

The past decade has seen remarkable improvements in our knowledge of this extinct order of birds. Within the context of this new generation of dinornithiform research, the present study marks the first attempt at understanding moa biomechanics. However, the present analysis deals with static loadings. Safety factors during locomotion are mediated not only through bone robusticity, but also by posture and behaviour. The use of multi-body dynamics analysis, grounded in neontological studies, is needed to illuminate the origins of the profound differences between leg structure in families of moa, and the trade-off between cursoriality and safety factors in flightless giant birds in general. Moreover, the now-routine specific identification and sexing of moa bones [47], combined with a multi-proxy approach to dietary analysis and biomechanical modelling, has the potential to further our understanding of species dispersal, foraging strategies and predator–prey interactions within the Dinornithiformes. Alongside *Aepyornis maximus*, *D. robustus* was one of the largest palaeognath birds to have ever existed. As such, understanding the biomechanical constraints associated with such extremes in body mass in Aves may provide further insights into terrestrial locomotion and limits to body size during the transition from non-avian theropods to modern birds.
